# Alpha2-Antiplasmin: The Devil You Don't Know in Cerebrovascular and Cardiovascular Disease

**DOI:** 10.3389/fcvm.2020.608899

**Published:** 2020-12-23

**Authors:** Satish Singh, Sofiyan Saleem, Guy L. Reed

**Affiliations:** Department of Medicine, University of Arizona-College of Medicine, Phoenix, AZ, United States

**Keywords:** alpha2-antiplasmin, ischemic stroke, pulmonary embolism, deep vein thrombosis, fibrinolysis, thrombosis, plasmin

## Abstract

Alpha2-antiplasmin (α2AP), the fast-reacting, serine protease inhibitor (serpin) of plasmin, was originally thought to play a key role in protection against uncontrolled, plasmin-mediated proteolysis of coagulation factors and other molecules. However, studies of humans and mice with genetic deficiency of α2AP have expanded our understanding of this serpin, particularly in disease states. Epidemiology studies have shown an association between high α2AP levels and increased risk or poor outcome in cardiovascular diseases. Mechanistic studies in disease models indicate that α2AP stops the body's own fibrinolytic system from dissolving pathologic thrombi that cause venous thrombosis, pulmonary embolism, arterial thrombosis, and ischemic stroke. In addition, α2AP fosters the development of microvascular thrombosis and enhances matrix metalloproteinase-9 expression. Through these mechanisms and others, α2AP contributes to brain injury, hemorrhage and swelling in experimental ischemic stroke. Recent studies also show that α2AP is required for the development of stasis thrombosis by inhibiting the early activation of effective fibrinolysis. In this review, we will discuss the key role played by α2AP in controlling thrombosis and fibrinolysis and, we will consider its potential value as a therapeutic target in cardiovascular diseases and ischemic stroke.

## Alpha2-Antiplasmin (α2AP) is the Serpin That Kills Plasmin

**Figure 1:**
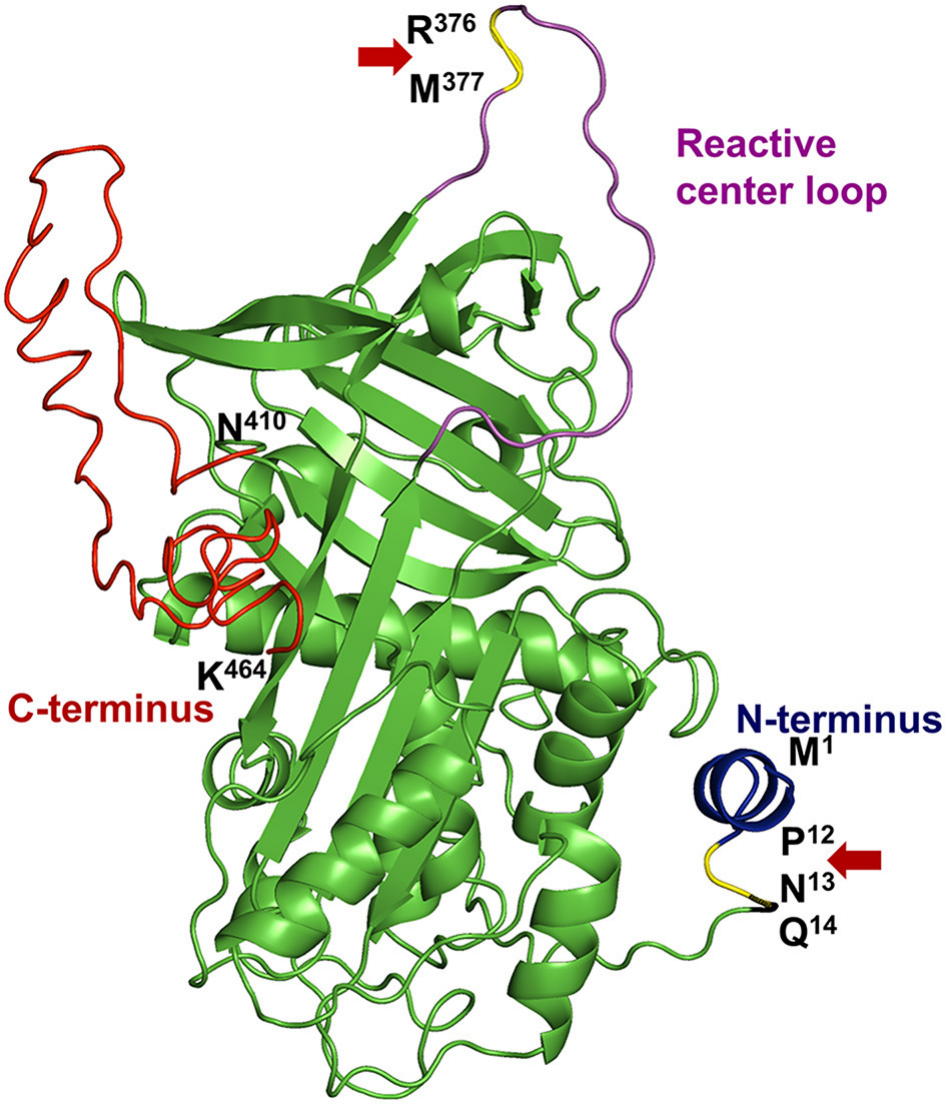
Structural elements of α2AP. The protein structure of human α2AP was generated by homology modeling using an online I-TASSER server (9) based on the crystal structure of mouse α2AP (PDB: 2R9Y) (10). α2AP has a central serpin domain and, N- & C-terminal extensions with specific functions. The N-terminus (blue) of α2AP is cleaved in the circulation by APCE at the Pro^12^-Asn^13^ site (P^12^-N^13^, yellow highlighted and Red arrow) to form Asn^13^-α2AP which represents 70% of blood α2AP levels. Gln^14^ (Q^14^) residue of α2AP is cross-linked to fibrin by transglutaminase enzyme, activated factor XIII. The reactive center loop (magenta) protrudes out from the central serpin domain (green) of α2AP. Plasmin cleaves at Arg^376^-Met^377^ bond (R^376^-M^377^, yellow highlighted, and red arrow) in the reactive center loop to form the inactive complex. A ~55 residue long C-terminus (red) (Asn^410^-Lys^464^; N^410^-K^464^) of α2AP is important for initial binding with plasmin due to the presence of multiple lysine residues.

α2AP (also known as α2-plasmin inhibitor, antiplasmin, serpinf2, plasmin inhibitor), is an ultrafast covalent inhibitor of plasmin (1–3) and, is a crucial member of the serine protease inhibitor (serpin) family. α2AP was first described by three different investigators as the fast-acting inhibitor of plasmin (4–6), who named it differently as α2-plasmin inhibitor (5), antiplasmin (6) and primary plasmin inhibitor (4, 7). α2AP is present in the blood at nearly half the concentration (~1 μM) of its target enzyme precursor, plasminogen (~2 μM) (6, 8). Structurally, α2AP is a unique serpin (Figure 1) with a 12 amino acid N-terminus, a central serpin domain and a C-terminal tail that is ~55-residues long (10–12). Mechanistically, the C-terminal lysine residues of α2AP initially bind non-covalently to the kringle domains of plasmin to form a 1:1 stoichiometric complex (13). Plasmin then cleaves the reactive center loop of α2AP at Arg^376^-Met^377^ bond and forms an inactive, covalent complex (1–3, 14). However, mutations in the α2AP molecule or monoclonal antibodies against α2AP can change the plasmin-α2AP interaction to an enzyme-substrate reaction (an alternate mechanism of serpin interaction) where active plasmin leaves the complex after cleaving α2AP (15).

## α2AP Expression

α2AP is primarily synthesized by hepatocytes in the liver and released into the blood (16, 17). After synthesis, α2AP is enzymatically modified in the circulation at both the N- and C-terminus, which affects its fibrin-crosslinking and plasmin(ogen) binding capabilities respectively (18). Lower levels of α2AP are also detected in the human kidney, blood platelets, the gastrointestinal tract, muscles, lungs, placenta, and brain (cerebral cortex, hippocampus, and cerebellum) (https://www.proteinatlas.org/ENSG00000167711-SERPINF2/tissue) (19– 21). α2AP is present among diverse species from mammals to birds and fish and there is significant protein sequence homology among various species such as humans, mice, bovine, etc. (11, 22–26). Human and mouse α2AP have similar kinetic constants for inhibition of autologous and heterologous plasmin *in vitro* (27). Administration of physiologic concentrations of human α2AP to α2AP-deficient (α2AP^−/−^) mice restores fibrinolytic inhibition and thrombosis to approximately normal levels (28). Since mouse and human α2AP have similar properties and cross-species reactivity, α2AP^−/−^ mice have provided an excellent translational model to examine the function of α2AP.

## α2AP and Control of Fibrinolysis

α2AP is covalently cross-linked to fibrin in the thrombus by activated factor XIII, a transglutaminase (29–31) which is a major source of the resistance of *in vitro* plasma clots to plasmin-mediated fibrinolysis (32–35). Once released into plasma, Met^1^-α2AP is clipped by α2AP cleaving-enzyme (APCE) at the N-terminus to generate the truncated Asn^13^-α2AP (Figure 1), which is incorporated into the fibrin network 13 times faster than uncleaved Met^1^-α2AP (36). Plasmin activity is partially protected from α2AP inhibition when its lysine binding sites are engaged with fibrin in the clot (37–39) or on the surface of a cell, such as endothelial cells (40). The relative contribution of activated factor XIII-mediated fibrin-fibrin cross-linking (41) vs. fibrin-α2AP crosslinking to thrombus resistance has been debated (35). Most of the studies suggest that fibrin-α2AP cross-linking is the major determinant of fibrinolytic resistance of the thrombus (34, 42–44). Under *in vivo* conditions, activated factor XIII also may contribute to the dynamics of thrombosis through secondary interactions such as red blood cell retention (45, 46), or cross-linking of other fibrinolytic inhibitors such as plasminogen activator inhibitor-1 (PAI-1) and thrombin-activatable fibrinolysis inhibitor (TAFI). *In vitro*, fibrinolysis assays showed that α2AP works synergistically with other major fibrinolytic inhibitors including TAFI or PAI-1 (47). *In vivo*, TAFI-deficient mice have variable effects in different pulmonary embolism models (48, 49). In contrast, α2AP^−/−^ mice showed greater fibrinolytic dissolution of *ex vivo* pulmonary thrombi as compared to PAI-1 deficient mice (50), suggesting that α2AP is the dominant contributor to thrombus resistance against fibrinolysis (50).

## Role of α2AP in Animal Models of Cardiovascular and Cerebrovascular Diseases

### Role of α2AP in Ischemic Stroke

Human ischemic stroke is primarily caused by thrombotic arterial occlusion of a middle cerebral artery which interrupts the supply of blood, oxygen and nutrients, leading to ischemia, inflammation, breakdown of the blood-brain barrier and neuronal cell death (51). Higher blood levels of α2AP are associated with an increased risk of human ischemic stroke and may contribute to the failure of recombinant-tissue plasminogen activator (r-tPA) therapy for reperfusion in stroke patients (52, 53).

**Figure 2:**
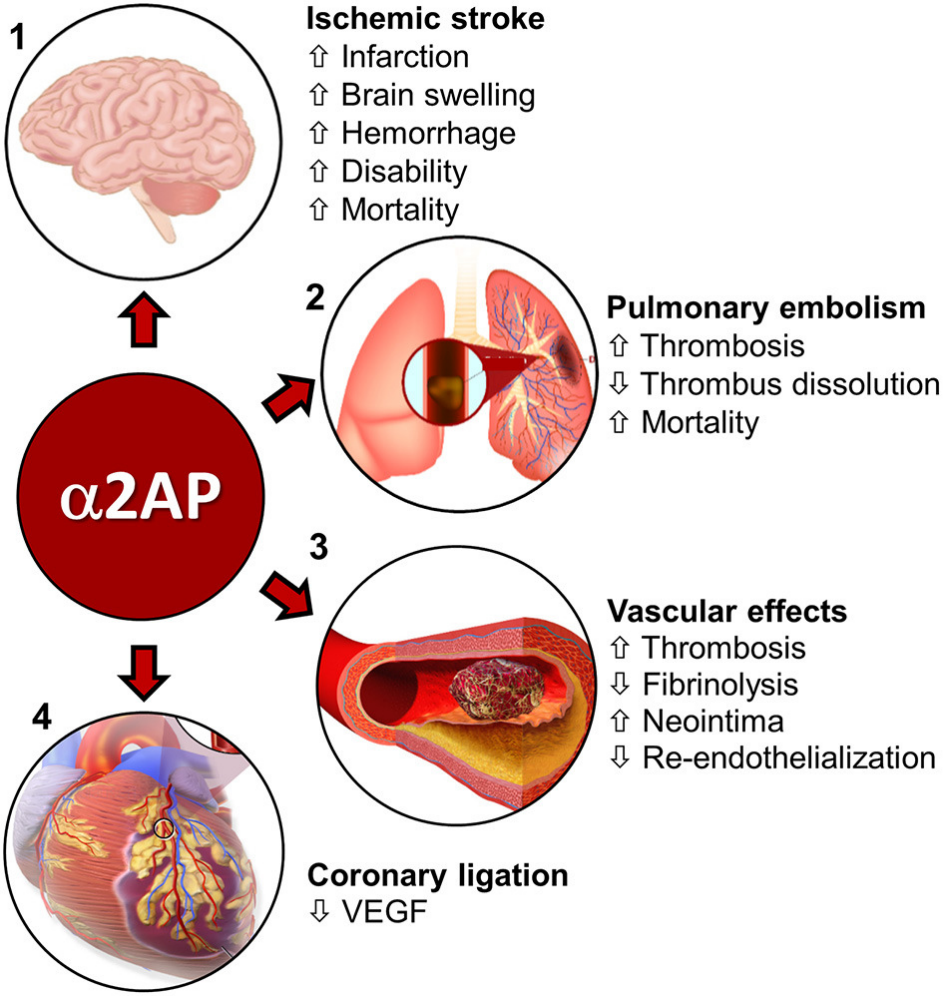
Role of α2AP in cardiovascular diseases. **(1)** Ischemic stroke—α2AP increases cerebral infarction, brain swelling, hemorrhage, and, disability and mortality during ischemic stroke in mice (55–59). Image source-(https://www.injurymap.com/free-human-anatomy-illustrations). **(2)** Pulmonary embolism-α2AP decreases thrombus dissolution and, increases thrombosis in the lungs and mortality during pulmonary embolism in mice (28, 60–62). Image source—(http://apsfa.org/pulmonary-embolism/). (http://www.nhlbi.nih.gov/health/dci/Diseases/pe/pe_what.html). **(3)** In arterial (carotid artery injury) and venous (IVC, jugular vein) injury/thrombosis models, α2AP increases thrombosis and decreases fibrinolysis (63, 64). α2AP is also associated with increased neointima formation and reduced endothelialization over time (65). Image source—(https://commons.wikimedia.org/wiki/File:Blausen_0088_BloodClot.png). **(4)** Coronary artery ligation—α2AP decreases VEGF levels in mice, which affect pulmonary vascular permeability (66). Image source—modified from (https://commons.wikimedia.org/wiki/File:Blausen_0463_HeartAttack.png).

Experimental studies show that α2AP regulates fibrinolysis during ischemic stroke and has deleterious effects that worsen brain injury by enhancing thrombo-inflammatory mechanisms (54). In a mouse model of thromboembolic occlusion of the middle cerebral artery, Houng et al. (55) showed that increased blood levels of α2AP reduced thrombus dissolution after treatment with r-tPA. Increased blood levels of α2AP also worsened brain infarction and brain swelling (55) (Figure 2). In contrast, α2AP inactivation (α2AP-I) enhanced r-tPA-mediated thrombus dissolution, in addition to reducing cerebral infarction, brain swelling and brain hemorrhage (55) (Figure 2). Treatment with the α2AP-I led to reduced TUNEL-staining, decreased caspase-3 expression and diminished breakdown of the blood-brain barrier. Subsequent studies showed that α2AP had dose-dependent deleterious effects in ischemic stroke in mice: it reduced thrombus dissolution and worsened cerebral infarction, brain swelling, and blood-brain barrier breakdown (56) (Figure 2). Increasing blood levels of α2AP enhanced ischemic brain injury, in part through a matrix-metalloproteinase-9 (MMP-9)-dependent mechanism (67). In contrast, both α2AP deficiency (α2AP^−/−^) or α2AP-I reduced brain infarction, hemorrhage and brain swelling (54–56). Also, α2AP deficiency or α2AP-I reduced microvascular thrombosis and MMP-9 expression (54, 56). Importantly, α2AP-I significantly enhanced acute (24 h) and longer-term (1 week) survival by comparison to r-tPA therapy and controls (54–56). Also, α2AP-I significantly improved neurobehavioral outcomes (54–56).

The effects of α2AP on ischemic stroke have also been studied in ischemic stroke, induced by mechanical arterial ligation or occlusion. Nagai et al. found that α2AP^−/−^ mice had reduced ischemic brain injury in comparison to control mice with normal α2AP levels after permanent ligation of the middle cerebral artery (57) (Figure 2). Similarly, inhibition of α2AP activity by intravenous infusion of plasmin/microplasmin or a monoclonal antibody significantly reduced focal ischemic brain injury after middle cerebral artery ligation in mice and hamsters (58). In ischemic stroke caused by middle cerebral artery photothrombotic occlusion, Suzuki et al. (68) found that doses of microplasmin that were equally effective for reducing ischemic cerebral infarction to r-tPA, caused less intracerebral bleeding and reduced tail bleeding time. However, higher doses of microplasmin that fully depleted circulating α2AP increased intracerebral bleeding (68, 69). It is interesting to note that α2AP deficiency or inhibition improved stroke outcomes in stroke models caused by an occluding thrombus and by mechanical ligation, suggesting the role of α2AP in stroke may extend beyond its role in thrombus dissolution.

### Role of α2AP in Deep Vein Thrombosis

Since its discovery, the effects of α2AP were considered to be restricted to inhibiting the dissolution of formed thrombi; it was not thought to have a role in regulating thrombus formation or thrombosis (70). However, new data show that α2AP regulates thrombus initiation and thrombus development, and is required for the occurrence of stasis induced deep vein thrombosis in mice (64) (Figure 2). In mice with normal levels of α2AP, thrombosis induces plasmin generation (64), however thrombosis proceeds because the plasmin generated, is insufficient to overcome the anti-fibrinolytic effects of α2AP. In contrast, in α2AP deficiency, plasmin-driven fibrinolysis prevents the initiation and establishment of thrombosis. Indeed, in venous thrombosis induced by stasis (no blood flow), or by stenosis (reduced flow), α2AP^−/−^ mice do not develop thrombosis even after hours to weeks (64). The requirement for α2AP to enable the development of thrombosis appears to be mediated through its inhibition of plasmin because another plasmin inhibitor, ε-aminocaproic acid (64) will restore thrombus formation in the absence of α2AP. How α2AP affects other key components of venous thrombosis such as neutrophils, monocytes or coagulation system components needs further investigation.

In a jugular vein thrombosis model (endothelial injury) in mice, α2AP deficiency caused delayed occlusion and early reperfusion in comparison to wild type controls (Figure 2) (63). An α2AP-I alone or in combination with r-tPA increased the dissolution of human plasma thrombi in a jugular vein thrombosis model in rabbits (71). The combination of r-tPA with the α2AP antibody did not increase fibrinogen degradation (71) suggesting that α2AP-I may enhance the specificity of fibrinolysis by plasminogen activators.

### α2AP in Pulmonary Embolism

Pulmonary embolism is caused when the thrombi formed in the deep veins of the legs or other sites detach from the vascular wall and travel to the lungs to cause serious, life-threatening complications (72). Therapy with r-tPA is limited to high-risk pulmonary embolism patients because clinical trials have shown r-tPA can cause serious or fatal bleeding (72). *In vivo* studies in mice and other animals have shown that thrombus dissolution can be achieved by α2AP-I with increased efficacy without increased bleeding. In a pulmonary embolism model in ferrets, α2AP-I by a monoclonal antibody increased experimental thrombus dissolution by r-tPA without increased fibrinogen degradation (60). Similarly, α2AP^−/−^ mice showed enhanced dissolution of pulmonary emboli made from α2AP^+/+^ or α2AP^−/−^ mouse plasma (61) (Figure 2), but in two different bleeding tests, α2AP^−/−^ mice did not show enhanced bleeding when compared to α2AP^+/+^ mice (61). Other hemostatic parameters including plasminogen, PAI-1 levels, hematocrit and fibrinogen levels were comparable in α2AP^−/−^ and α2AP^+/+^ mice (61). The role of α2AP was also studied in a pulmonary thrombosis model induced by photochemical irradiation of Rose Bengal in the jugular vein in α2AP^−/−^ mice (73) (Figure 2). In this model, α2AP deficiency was associated with decreased deposition of endogenous fibrin in pulmonary vessels and increased survival in comparison to wild type controls (α2AP^+/+^) (73). There were no differences in the bleeding time in α2AP^−/−^ mice treated by r-tPA (73). Inhibition of the crosslinking of α2AP to fibrin by activated factor XIII markedly enhanced fibrinolysis in experimental pulmonary thromboembolism (44). Finally, the comparative effects of an α2AP-I and r-tPA were examined in a humanized model of pulmonary embolism in mice. The α2AP-I alone showed comparable efficacy to high dose r-tPA in thrombus dissolution (28) (Figure 2). Treatment with r-tPA increased fibrinogen consumption and prolonged bleeding times but α2AP-I did not cause these effects. Combination treatment with very low dose r-tPA and α2AP-I was more effective at dissolving thrombi than a much higher dose of r-tPA alone, but the combination did not cause increased fibrinogen degradation and/or prolonged bleeding time (28) (Figure 2).

### α2AP in Arterial Injury and Thrombosis

The role of α2AP in arterial thrombosis was investigated by Matsuno et al. (63) by inducing endothelial injury of the murine carotid artery (Figure 2). α2AP deficiency did not change the time for thrombotic occlusion of the carotid artery but it significantly accelerated spontaneous reperfusion indicating that α2AP played a major role in arterial fibrinolysis (63). In studies of femoral arterial injury induced by electric current, α2AP did not appear to affect smooth muscle cell migration and neointima formation 2–3 weeks after injury (74). However, in carotid artery injury induced by Rose Bengal photo-irradiation, there was increased re-endothelialization and reduced neointima formation in α2AP^−/−^ mice in comparison with α2AP^+/+^ mice (65) (Figure 2). The increased re-endothelialization was attributed to the increased plasmin-mediated generation of vascular endothelial growth factor in α2AP^−/−^ mice (Figure 2). Finally, Ang II and N(omega)-nitro-L-arginine methyl ester (L-NAME)-induced vascular remodeling (perivascular fibrosis) was significantly decreased in α2AP^−/−^ mice compared with wild-type mice (75).

### α2AP in Coronary Artery Ligation

Coronary thrombosis is the primary cause of human myocardial infarction (76) but a reproducible model of coronary thrombosis has not been established in mice (77), which limits the translational relevance of experimental studies. Nevertheless, in a left coronary artery permanent ligation model in mice, α2AP deficiency was associated with increased plasmin-mediated vascular endothelial growth factor release, which enhanced pulmonary vascular permeability (Figure 2). Unfortunately, the effects of α2AP deficiency on thrombosis or fibrinolysis could not be assessed in this model. The role of α2AP in an ischemia-reperfusion model of myocardial infarction has not been studied.

### α2AP in Thrombotic Thrombocytopenic Purpura

Thrombotic thrombocytopenic purpura (TTP) is a rare but severe thrombotic disorder causing microvascular thrombosis in various organs and low platelet counts (78). In ADAMTS deficiency-induced experimental TTP in mice, there are increased levels of von Willebrand factor in the blood and microthrombi. However, increased plasmin activity by α2AP deficiency in mice causes increased proteolysis of von Willebrand factor and resolves the signs of disease (79).

## Non-Fibrinolytic Effects of α2AP

α2AP may have non-fibrinolytic effects under different pathophysiological conditions. α2AP deficiency in mice decreases fibrosis in different models of fibrotic diseases (80, 81). Cancer is one of the major risk factors for deep vein thrombosis and α2AP enables deep vein thrombosis (64); however, it also restricts lymphatic remodeling and metastasis in a mouse model of cancer (82). In the brain, α2AP is expressed mainly by hippocampal neurons and is required for dendrite growth through p38 microtubule-associated protein kinase pathways in mice (83, 84). α2AP deficiency has been associated with impairment in motor function, cognitive function, anxiety, and depression-like behavior in mice (85). In a mouse model of Alzheimer's disease, chronic depletion of blood α2AP by antisense oligonucleotide treatment increased the activation of macrophage/microglial cells and increased fibrillar plaque, though it did not alter total plaque deposition (86). α2AP deficiency accelerates wound healing, perhaps through an increase in the release of vascular endothelial growth factor (87). Inhibitors of mouse α2AP increase liver repair after injury when compared to controls (88). α2AP deficiency also decreases arteriosclerosis after vascular injury (65).

## Deficiency of α2AP in Humans—Congenital and Acquired

Congenital deficiency of α2AP (Miyasato disease) in humans is very rare and has been associated with a phenotype of delayed traumatic or spontaneous rebleeding, usually in the form of hematomas or hemarthroses (89–91). Spontaneous cerebral bleeding has not been reported as an issue in humans (89–91). Bleeding in α2AP deficiency is usually controlled by standard measures or with tranexamic acid or ε-aminocaproic acid, which block plasmin-mediated fibrinolysis (91). Indeed, α2AP-deficient patients have successfully undergone heart surgery with these agents. Homozygous genetic deficiency has been described in a 62-year-old patient (92), indicating that the life-long absence of α2AP can be tolerated. Heterozygous individuals normally do not show bleeding phenotype unless there is a trauma or surgery; and sporadic reports of α2AP heterozygous deficiency in patients as old as 83 years are reported (93).

Congenital deficiency can be either quantitative with reduced protein levels or qualitative with reduced protein function but both are difficult to detect as routine coagulation tests and other hemostatic parameters are normal in patients. Quantitative deficiency of α2AP with reduced protein levels may be caused by a point mutation (α2AP-Paris Trousseau, 15% levels, and α2AP Val^384^-Met, ~50% levels), or a deletion (α2AP-Okinawa, <1%) or a frameshift mutation (α2AP-Nara, <1% level) (91). A qualitative or functional deficiency of human α2AP (Enschede) is due to an insertional mutation in the reactive center loop of α2AP (an additional alanine), which causes it to behave like a substrate of plasmin instead of an inhibitor (94, 95).

Acquired deficiency of α2AP may be caused by thrombolytic agents (e.g., plasminogen activators, plasmin, and microplasmin) or disease conditions such as severe liver disease and acute leukemia (54). Increased levels of α2AP are associated prospectively with an elevated risk of myocardial infarction (96) and ischemic stroke (52). In Alzheimer's disease patients, α2AP expression increases in the brain tissue and is associated with amyloid β plaques (20). During early studies of plasminogen activators, levels of α2AP were noted to fall before or synchronously with fibrinogen levels, which was a harbinger of clinical bleeding complications (97, 98). Indeed, α2AP supplementation was considered as an adjuvant to r-tPA therapy to prevent bleeding complications (99). However, more recent studies show that α2AP is the dominant inhibitor of physiologic fibrinolysis and that elevated levels of α2AP may be harmful in cardiovascular and cerebrovascular diseases (1, 26).

## Therapeutic Strategies Targeting α2AP

Thrombosis is the leading cause of cardiovascular and cerebrovascular deaths (100). There have been two primary strategies for treating thrombotic diseases: anticoagulation to prevent thrombus formation or expansion, and fibrinolytics to dissolve existing thrombi. Anticoagulation therapy is widely used to prevent thrombosis in patients with myocardial infarction, ischemic stroke, deep vein thrombosis and pulmonary embolism (51, 72, 101). The value of anticoagulation is limited by bleeding and by the fact that it does not dissolve existing thrombi. Fibrinolytic (thrombolytic) therapy triggers the dissolution of existing thrombi. Plasminogen activators such as r-tPA, tenecteplase, and streptokinase are the most widely used fibrinolytic agents. The value of plasminogen activator therapy is limited by bleeding and other toxicities, which restrict therapy to a small subset of those who might benefit from thrombus dissolution for treatment of ischemic stroke, myocardial infarction, pulmonary embolism, etc. (51, 72) Experimental studies suggest that targeting α2AP is a novel paradigm for preventing thrombosis and dissolution of thrombi without compromising safety. Several strategies have been described including specific monoclonal antibodies, peptides, and microplasmin to neutralize the activity of α2AP.

### Monoclonal Antibodies Inhibiting α2AP Activity

Reed et al. (62, 71, 102) and Sakata et al. (103) reported the use of monoclonal antibodies to inhibit human α2AP activity to enhance thrombus dissolution. Mouse monoclonal antibodies caused spontaneous or r-tPA-mediated human clot dissolution (33, 62, 102, 103). A mouse monoclonal antibody inhibitor of α2AP synergistically increased fibrinolysis by r-tPA and other types of plasminogen activators increasing the potency of these agents by 20–80-fold (62). Despite increases in fibrinolysis, equipotent combinations of α2AP-I with very low dose plasminogen activators caused less fibrinogen breakdown than the plasminogen activator alone. As noted earlier, α2AP-I has been shown to enhance fibrinolysis in several different animal models of venous thrombosis, pulmonary embolism and ischemic stroke (28, 44, 55, 56, 59, 60, 62, 64, 71). More recently, in a humanized model of pulmonary embolism in mice, an α2AP-I (TS23, a monoclonal antibody that inactivates human α2AP), enhanced the dissolution of pulmonary emboli with a potency similar to higher dose r-tPA (3 mg/kg), though unlike r-tPA, this α2AP-I did not increase arterial or venous bleeding (28). The α2AP-I, TS23 prevented thrombus formation during venous stasis in mice (64). This α2AP-I has been tested in Phase I trials in humans (NCT03001544) and Phase II trials are planned.

### Microplasmin/Plasmin

Microplasmin is a truncated version of plasmin that contains only the catalytic domain (104). Microplasmin is a non-specific enzyme that is inhibited by α2-macroglobulin and by α2AP. Infusions of microplasmin will induce secondary depletion of α2AP, which were thought to be important for its function. A single bolus of plasmin/microplasmin in mice significantly reduced focal ischemic injury in mice (58). Microplasmin also reduced ischemic brain injury and neurological function in a rat middle cerebral artery thrombosis model (105) and improved behavioral outcomes in an embolic stroke model in rabbit (106). A Phase 1 trial in humans showed that α2AP inhibition by microplasmin induced a dose (0.1–5 mg/kg) related inhibition effect on α2AP activity in healthy volunteers (107). In a double-blind randomized phase II trial in stroke patients, 1–4 mg/kg microplasmin neutralized blood α2AP by up to 80% and was well-tolerated, however, no effect on reperfusion or clinic outcome was observed possibly due to the small sample size (108). The development of microplasmin as a cardiovascular therapeutic was discontinued and, is used now for the clinical treatment of human retinal disease (109).

Infusion of plasmin will also deplete α2AP and this was used as an experimental treatment for thrombotic injury in mice (58). Marder et al. (110) used a similar strategy with a different hypothesis namely, that catheter-directed localized delivery of plasmin will increase the thrombus dissolution and then the released plasmin will be neutralized by α2AP in circulation so that plasmin will not have any side effects (111). Plasmin (4 mg/kg) dissolved thrombi in abdominal aorta thrombosis and did not increase bleeding (112). Plasmin of up to a dose of 8 mg/kg completely neutralized 60% of α2AP activity but also caused fibrinogen, factor VIII depletion, as well as increased bleeding (113). Safety trials for plasmin in patients with acute lower extremity arterial or bypass graft occlusion showed enhanced thrombus dissolution with bleeding events in <20%(114). Phase I/II of human plasmin in acute ischemic stroke patients showed that human plasmin was tolerable for plasmin dose up to 80 mg within 9 h of stroke, but recanalization was achieved in a limited number of patients (25%) (115). There have been no new reports of clinical development of plasmin.

### Inhibitors of APCE

APCE is a 97 kDa, prolyl-specific protease in plasma that cleaves Met^1^-α2AP at Pro^12^-Asn^13^ to generate Asn^13^-α2AP, which is cross-linked to fibrin 13 times faster than Met^1^-α2AP (36). APCE shares a strong amino acid sequence homology to fibroblast activation protein, an integral transmembrane protein and may represent its soluble isoform or a derivative (36). It was proposed that the specific inhibitors of APCE can reduce the amount of α2AP crosslinking to fibrin and thus enhance fibrinolysis. Chemically modified peptide inhibitors of APCE increased fibrinolysis in plasma clot lysis assays (116, 117).

### α2AP Mimicking Peptides

Synthetic peptide mimicking α2AP regions have been tested as a competitive inhibitor of α2AP to interfere in factor XIII-mediated cross-linking, plasminogen binding and activation to achieve enhanced fibrinolysis or clot lysis *in vitro* (118–121). The effects of these peptides or inhibitors have been studied during clot formation, but not on preformed plasma clots or *in vivo* thrombi in experimental models.

## Summary

Several different approaches have been taken to investigate the therapeutic potential of interfering with α2AP function to prevent thrombosis and dissolve existing thrombi. Systemic use of microplasmin has been limited by off-target effects and its use is currently limited to the treatment of retinal disease. Plasmin administration required catheter delivery by expert teams and only achieved limited thrombotic dissolution and recanalization in ischemic stroke. The development of α2AP mimicking peptides and APCE inhibitors appears uncertain as there are no reports of clinical trials. Monoclonal antibody approaches have been extensively evaluated in experimental models; they have shown high specificity, potency and the fewest off-target effects and are in development for Phase II trials in thrombotic diseases.

## Concluding Remarks and Future Perspectives

Since its original description by three different laboratories in 1976, our understanding of α2AP and its role in cardiovascular has evolved significantly. Epidemiologic and observational studies suggest that α2AP contributes significantly to the risk of thrombotic events in cardiovascular and cerebrovascular diseases. Numerous *in vitro* and *in vivo* studies, including studies in genetically-deficient mice and humans indicate that α2AP regulates endogenous and pharmacologic fibrinolysis. In addition, α2AP has been implicated in experimental models of wound healing, fibrosis, neuronal function, liver repair, and Alzheimer's disease. Disease-relevant models of thrombosis have shown that blocking α2AP function significantly enhances thrombus dissolution and improves outcomes, without causing bleeding. Taken together, these data suggest that therapeutically targeting α2AP has promise for both treatment and prevention of acute thrombotic cardiovascular and cerebrovascular diseases.

## Data Availability Statement

The original contributions presented in the study are included in the article/supplementary material, further inquiries can be directed to the corresponding author/s.

## Author Contributions

SSa edited the manuscript. SSi and GR wrote, edited, and approved the manuscript. All authors contributed to the article and approved the submitted version.

## Conflict of Interest

GR is the founder of Translational Sciences. The remaining authors declare that the research was conducted in the absence of any commercial or financial relationships that could be construed as a potential conflict of interest.
